# QSAR-based rational discovery of novel substituted-4′-iminospiro[indoline-3,3′-[1,2,5]thiadiazolidinyl]-2-one 1′,1′-dioxide with potent in vitro anticancer activity

**DOI:** 10.1186/s13065-019-0520-z

**Published:** 2019-01-29

**Authors:** Mohammed A. Khedr, Reem I. Al-Wabli, Maha S. Almutairi, Wafaa A. Zaghary

**Affiliations:** 1 Department of Pharmaceutical Chemistry, Faculty of Pharmacy, Helwan University, Ein Helwan, Cairo, 11790 Egypt; 2 Department of Pharmaceutical Chemistry, College of Pharmacy, King Saud University, Riyadh, 11451 Saudi Arabia; 3 Department of Pharmaceutical Sciences, College of Clinical Pharmacy, King Faisal University, Al Hasa, 31982 Saudi Arabia

**Keywords:** QSAR, Conformational rigidification, Molecular docking, Molecular dynamics, In vitro anticancer activity

## Abstract

Recent studies have suggested that aldose reductase inhibition preferentially inhibits the growth of cancer cells. However, the investigations of this issue are not many. Novel nine substituted- 4′-iminospiro[indoline-3,3′-[1,2,5] thiadiazolidinyl]-2-one 1′,1′-dioxide derivatives were designed by both isosteric replacement of the imidazolidine-2,5-dione moiety in spirohydantoin scaffold and conformational rigidification approaches. A QSAR with high predictive power (r^2^ = 0.99) was created from a series of potent aldose reductase inhibitors and was used to predict the activity of our new compounds. Compound 5 showed the best docking score (− 33.24 kcal/mol) with the least RMSD value (< 1.5) obtained by molecular dynamic simulations over 20 ns. All compounds showed promising anticancer activities especially compound 5 that achieved the highest inhibitory activities with IC_50_; 0.013, 0.031, 0.064, and 0.048 mmol/L against breast, colon, prostate, and lung cell lines respectively. The discovery of this lead compound confirmed the rational design. Further investigations may be required for optimization of this compound
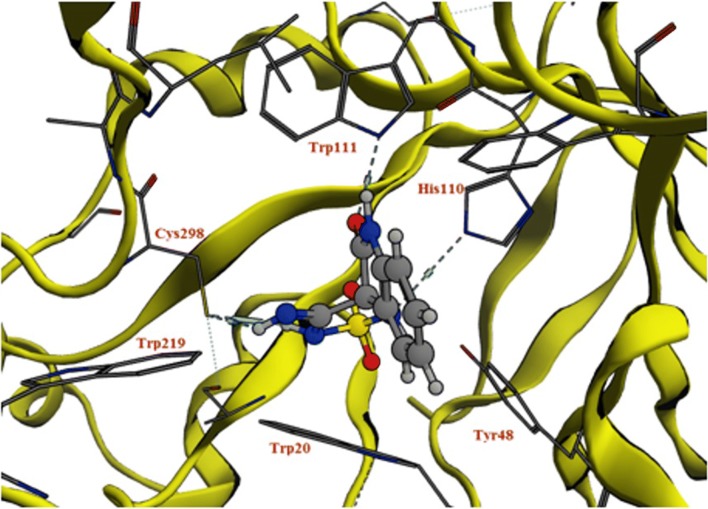
.

## Introduction

**Fig. 1:**
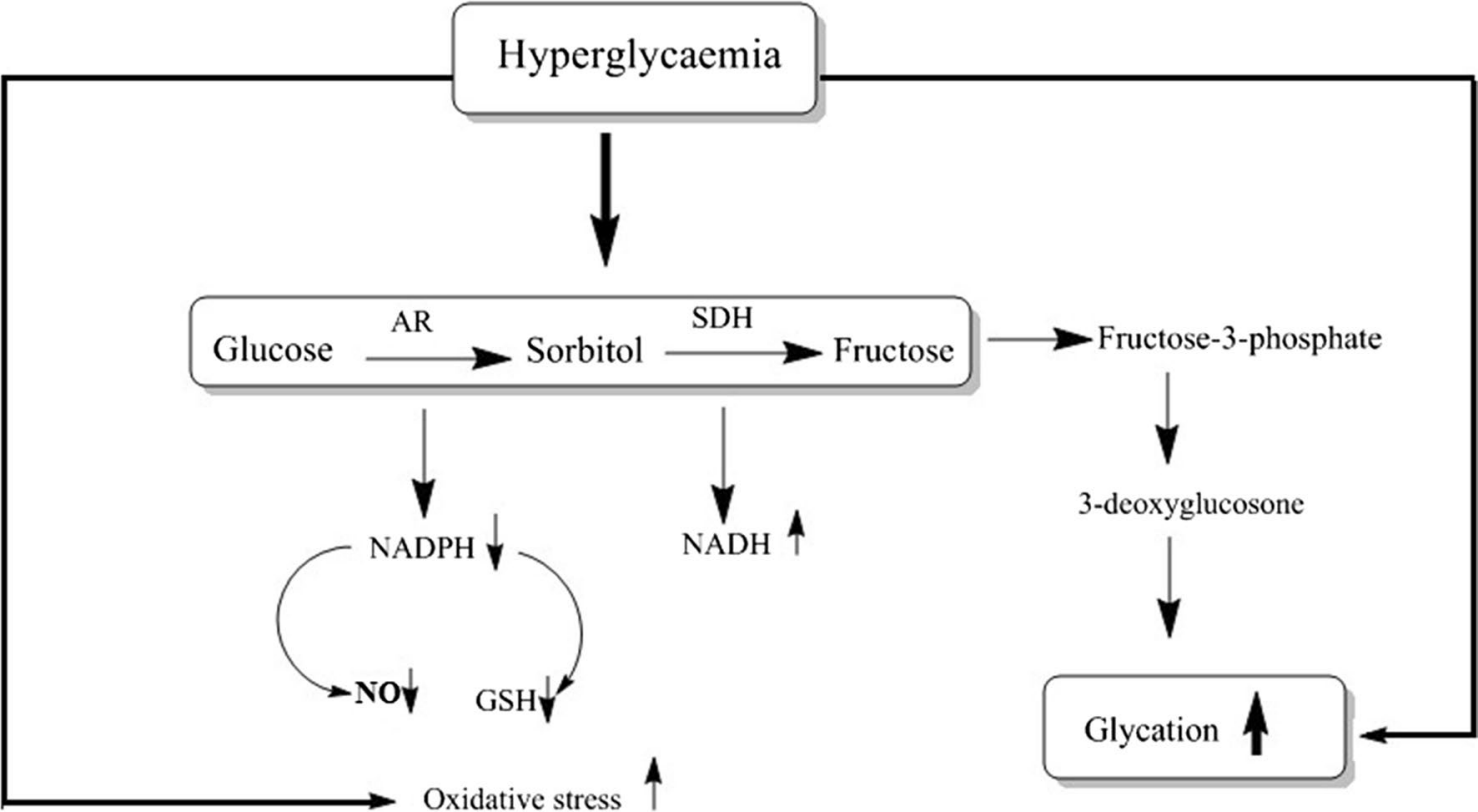
Polyol pathway showing the conversion of glucose into sorbitol

Aldose reductase (AR) is an enzyme that is found in mammalian tissues [1, 2]. Its main role is to convert glucose into sorbitol that will be then converted to fructose by the action of sorbitol dehydrogenase through the polyol pathway (Fig. 1); this process takes place in presence of high blood glucose levels. The activation of this pathway results in many complications as a result of sorbitol accumulation [3]. Chronic inflammation can be resulted from oxidative stress which in turn can be a main cause of cancer [4–7]. Aldose reductase inhibitors have different reported pharmacological actions such as: prevention of diabetic induced cataracts [8], AR inhibitors have been used as potential therapy in heart failure and ischemia [9], they also can prevent the ovarian dysfunction [10].

Recently, the AR inhibition was reported to prevent the growth factors’ activation in colon cancer [11]. The selective AR inhibition can also contribute in inhibition of cancer metastasis [12, 13]. Trials to develop AR inhibitors started after the isolation of the first enzyme in 1965 [14]. Systematic screening of some aliphatic carboxylic acids and some α,ω-dicarboxylic acids revealed some lead compounds with maximum AR inhibitory activity of 65% [15].

**Fig. 2:**
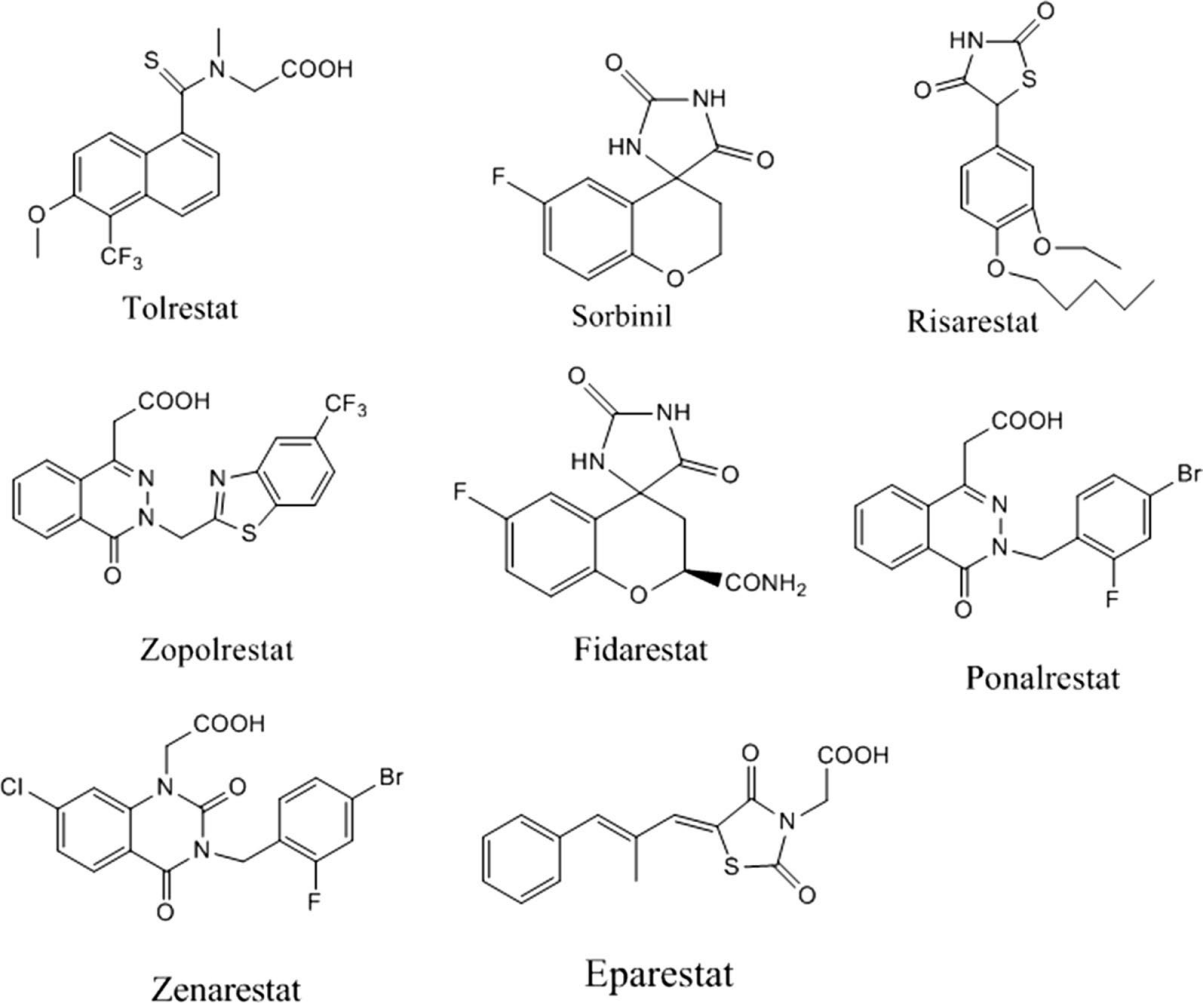
Structures of AR inhibitors drugs with different chemical scaffolds

Alrestatin was the first real AR inhibitor with inhibitory activity at dose of 250–300 mg/kg (potency). Screening of other series of carboxylic acids-containing compounds showed that phthaloylglycine was a potent AR inhibitor (IC_50_ = 2.5 × 10^−6^ M) however, it was orally inactive. In 1978, Pfizer discovered some spirohydantoin AR inhibitors with imidazolidine-2,5-dione scaffold like Sorbinil (IC_50_ = 0.5 μM) (Fig. 2) [16, 17]. Many derivatives of Sorbinil were then introduced with different halo substitution (Fig. 2) where the fluoro atom of sorbinil was replaced with 6,7-dichloro and 6,8-dichloro derivatives that were more potent than Sorbinil [18]. The overexpression of AR was observed in different cancerous tissues such as: colon, lung, breast and prostate [19]. The problem of this overexpression was associated with the tumor recurrence even after treatment [20]. Inhibition or deletion of AR showed a significant inhibition of growth factors, cytokines and chemokines that are involved in cancer [21]. The AR inhibition can be of great importance for cancer treatment [22].

Unfortunately, the anticancer studies of most AR inhibitors are not many. In addition, some of AR inhibitors like Fidarestat failed in clinical trials phase III due to some problems of hypersensitivity and requirement of high dose [23]. The aim of this work was to design and discover some novel compounds that can be potential AR inhibitors with expected anticancer activities via using a quantitative structure activity relationship (QSAR)—based approach.

## Results and discussion

Different heterocyclic moieties other than the spirohydantoin were developed as AR inhibitors. However, they were less active than spirohydantoin derivatives. Recently, novel potent AR inhibitors with cyano(2-oxo-2,3-dihydroindol-3-yl) acetic acid scaffold (scaffold A) were discovered [24]. These inhibitors showed more inhibitory activity than the spirohydantoin derivatives (Table 1). The AR inhibitory activity of other potent series of 2-oxo-2,3-dihydroindol derivatives were reported as well that confirmed the high affinity of the 2-oxo-2,3-dihydroindol scaffold toward binding with AR pockets [25]. However, the anticancer activity of this class was not investigated as well.

**Table 1:** 5-Substituted -cyano(2-oxo-2,3-dihydroindol-3-yl)acetic acid (scaffold A) derivatives with potent AR inhibitory activities

Compound	X	R	IC_50_ μM	*p*IC_50_
I	H	H	0.85	6.070
II	F	H	0.28	6.552
III	Br	H	0.38	6.420
IV	NO_2_	H	1.2	5.920
V	H	CH_2_-C_6_H_5_	3.2	5.494
VI	H	CH_2_-C_6_H_4_-*P*-F	0.57	6.244
VII	H	CH_2_-C_6_H_4_-*P*-CF_3_	0.57	6.244
VIII	H	CH_2_-C_6_H_4_-*P*-CH_3_	0.64	6.193
IX	H	CH_2_-C_6_H_4_-*P*-OCH_3_	0.63	6.200
X	F	CH_3_	0.39	6.408
XI	F	CH_2_-CH_2_-Cl	1.34	5.872
XII	F	CH_2_-CH_2_-CH_3_	0.28	6.552
XIII	F	CH_2_-C_6_H_5_	0.18	6.744
XIV	F	CH_2_-C_6_H_4_-*P*-F	0.13	6.886
XV	F	CH_2_-C_6_H_4_-*P*-CF_3_	0.28	6.552
XVI	F	CH_2_-C_6_H_4_-*P*-CH_3_	0.075	7.124
XVII	F	CH_2_-C_6_H_4_-*P*-OCH_3_	0.11	6.958
XVIII	Br	CH_2_-C_6_H_4_-*P*-CH_3_	0.25	6.602
Sorbinil			0.50	6.301
Fidarestat			0.15	6.823

### QSAR-based model AR inhibitors

The first step of our work involved the building of a QSAR model for AR inhibitors by using the most active series that has (scaffold A). The main aim of this step was to derive all-important descriptors that contribute to the AR inhibition. The QSAR model can be used for prediction of the biological activity of newly designed compounds with unknown activity.

### Identification of the correlated molecular descriptors

The main aim of the QSAR is the analysis of the experimental data and building of a numerical model to be used for further prediction. All *p*IC_50_ values for all derivatives were calculated (Table 1). It was important to identify the molecular descriptors that contribute to the biological activity. Molecular descriptors are molecular features that can be two dimensional descriptors that included: 14 physicochemical descriptors, 18 subdivided surface area, 41 atoms and bond counts, 15 adjacency and distance matrix descriptors, and 30 potential chare descriptors. In addition, the three dimensional descriptors that include 15 descriptors for potential energy, 21 Molecular Orbital Package (MOPAC) descriptors, 30 surface area and volume descriptors, and 18 conformation dependent descriptors.

Evaluation of each set of the previously mentioned descriptors was done by using regression analysis to find out which set has the best correlation with biological activity. An ideal set of descriptors will generate a model with least root mean square error (RMSE), and a r^2^ coefficient value close to 1.00. The cross validation step was also performed to evaluate the predicted *p*IC_50_ and to compare it to the real values. The residuals and *Z*-scores were also computed where any *Z*-scores > 2.5 were considered as outliers for a model and was excluded.

**Fig. 3:**
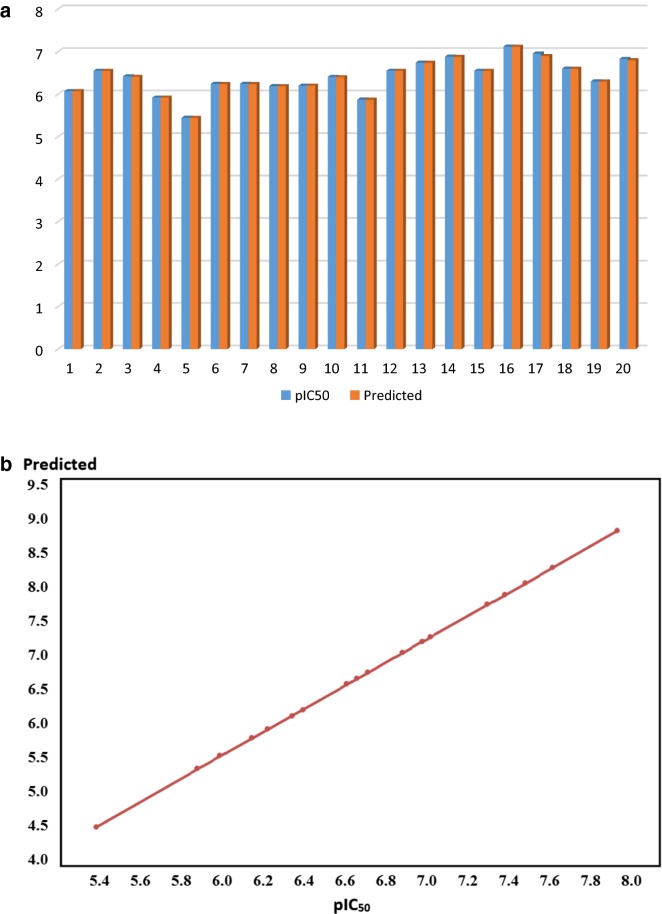
**a** The correlation between the *p*IC_50_ and predicted *p*IC_50._ **b** Linear relation plot indicating the proper selection of descriptors

From all the tested sets of descriptors, the 3D set that includes volume and shape descriptors was expected to be the best, where RMSE was 0.0032, the r^2^ coefficient was 0.99 and the *Z*-score was < 2.5 (Table 2). According to the results, the predicted *p*IC_50_ values were very close to the real calculated ones. A graphical correlation plot between the experimental *p*IC_50_ and the expected *p*IC_50_ values was generated (Fig. 3). The plot showed a linear relationship where, all values were on the same line.
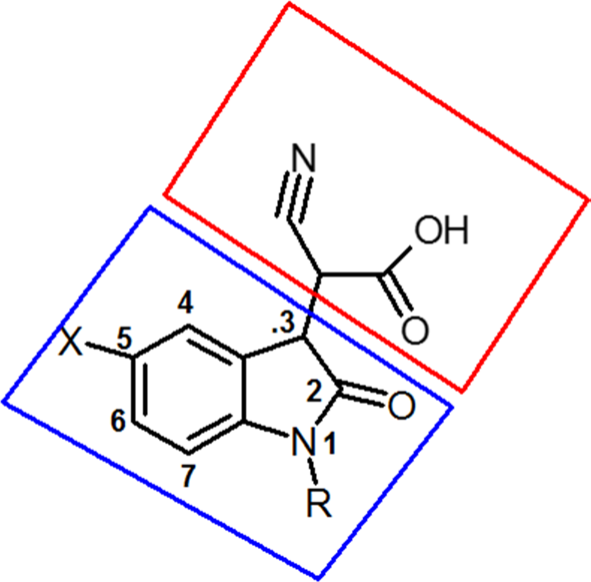

**Table 2:** Calculated *p*IC_50_, *Z*-score, and predicted *p*IC_50_ for the data set for building the QSAR model

Compound	IC_50_ (μM)	*p*IC_50_	Residual	*Z*-score	Predicted *p*IC_50_
I	0.85	6.070	0.0000	0.1116	6.07
II	0.28	6.552	0.0001	0.1684	6.55
III	0.38	6.420	0.0001	0.2929	6.42
IV	1.2	5.920	0.0001	0.2873	5.92
V	3.2	5.494	0.0001	0.4187	5.44
VI	0.57	6.244	0.0005	1.6129	6.24
VII	0.57	6.244	0.0006	1.7125	6.24
VIII	0.64	6.193	0.0007	2.2621	6.19
IX	0.63	6.200	0.0003	0.8417	6.20
X	0.39	6.408	0.0003	0.9366	6.40
XI	1.34	5.872	0.0000	0.0055	5.87
XII	0.28	6.552	0.0004	1.1437	6.55
XIII	0.18	6.744	0.0001	0.4482	6.74
XIV	0.13	6.886	0.0005	1.3919	6.88
XV	0.28	6.552	0.0004	1.1742	6.55
XVI	0.075	7.124	0.0004	1.1085	7.12
XVII	0.11	6.958	0.0003	0.5351	6.96
XVIII	0.25	6.602	0.0002	1.0190	6.60

### Validation of the QSAR model

Further validation was done by estimation of predicted *p*IC_50_ of two references drugs sorbinil and fidarestat as a test set. The predicted results were also highly close to the real values. The *Z*-score for all compounds in the training and test sets was less than 2.5. In addition, the residual values were neglected which indicated good and accepted model.

**Table:** 

Compound	IC_50_ (μM)	*p*IC_50_	Residual	*Z*-score	Predicted *p*IC_50_
Sorbinil	0.50	6.30	0.0000	0.0105	6.30
Fidarestat	0.15	6.82	0.0000	0.0072	6.80

### Design of novel scaffold

**Fig. 4:**
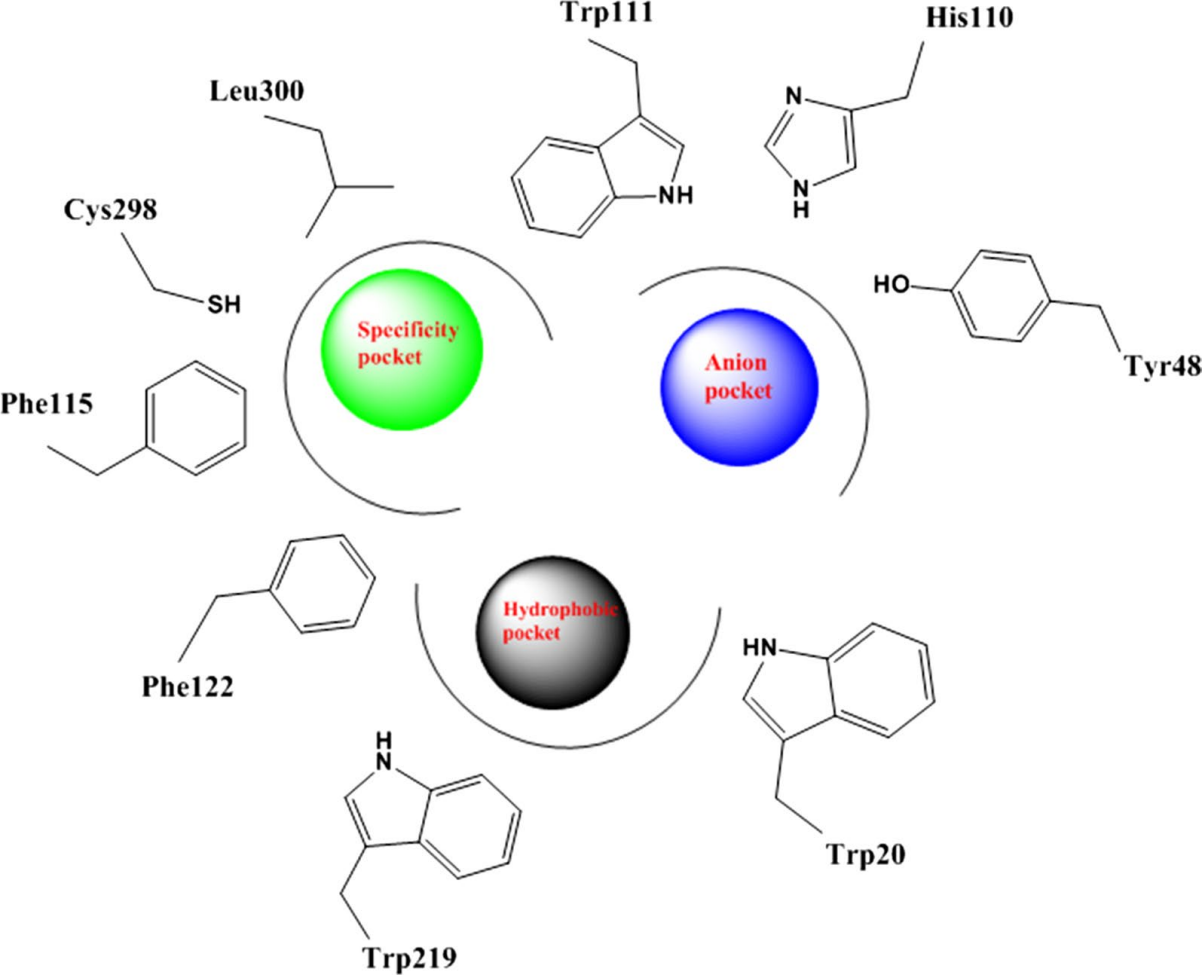
Ligand-binding pocket of human AR showing the main three pockets

Protein data bank was used to download the crystal structure of human AR in complex with Fidarestat (pdb code = 1ef3). The analysis of the ligand-binding pocket of AR crystal structure showed that it has three pockets namely; specificity pocket, anion pocket and hydrophobic pocket (Fig. 4).

**Fig. 5:**
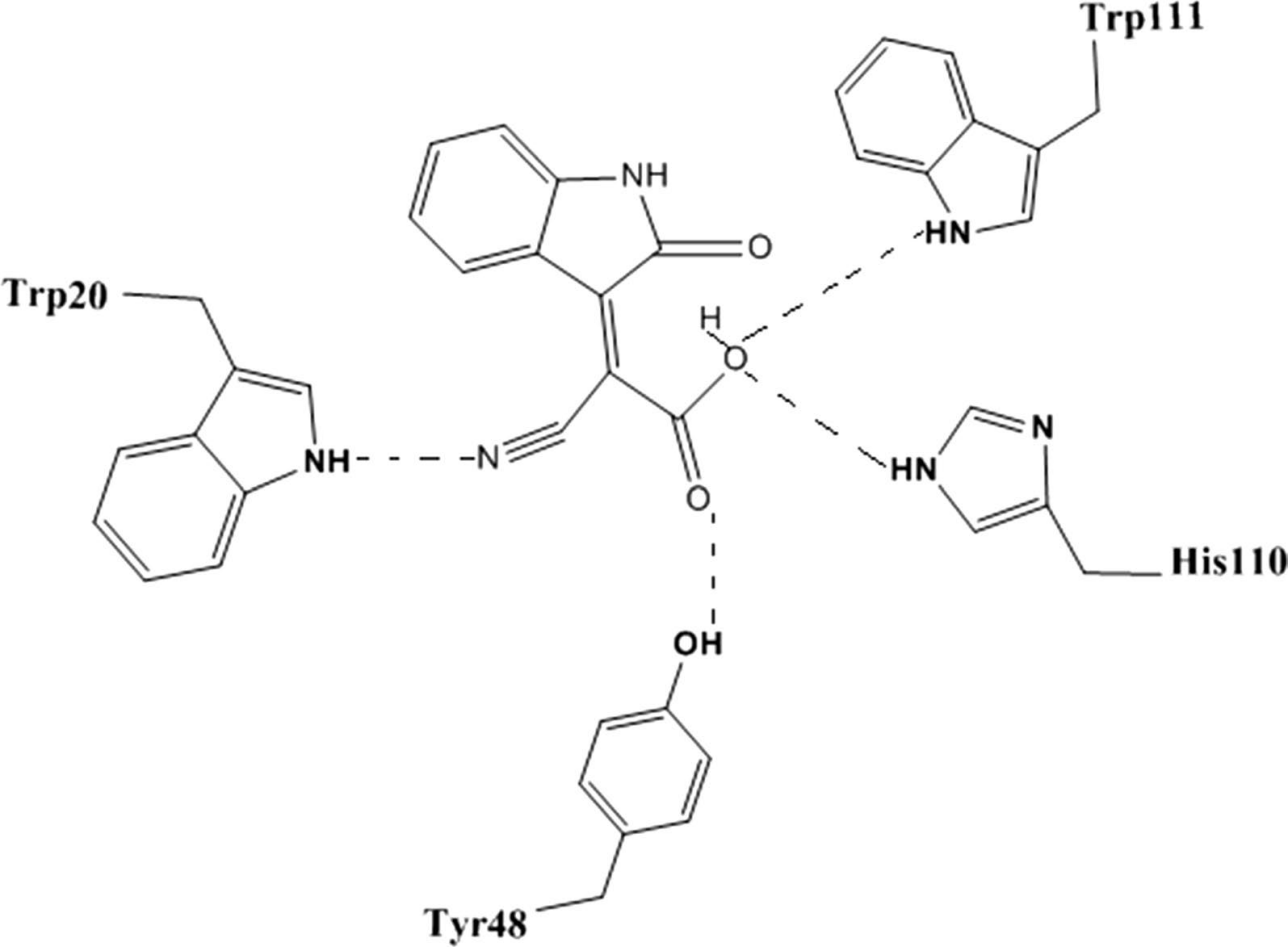
Predicted orientation mode of cyano(2-oxo-2,3-dihydroindol-3-yl) acetic acid scaffold

Scaffold A was studied for its predicted mode of orientation within these pockets. It was oriented between the specificity and anion pockets (Fig. 5) [24]. The presence of two acceptor oxygen atoms of carboxylic group and the nitrile group enabled them to form a network of hydrogen bonding with Trp111 (–NH), His110 (–NH), Tyr48 (–OH), and Trp20 (–NH). The hydrophobic moiety of the scaffold (2-oxo-2,3-dihydroindol-3-yl) was oriented between the two hydrophobic amino acids Trp111 and Trp20 that strengthened the π–π interactions.

### Pre-molecular docking study

**Fig. 6:**
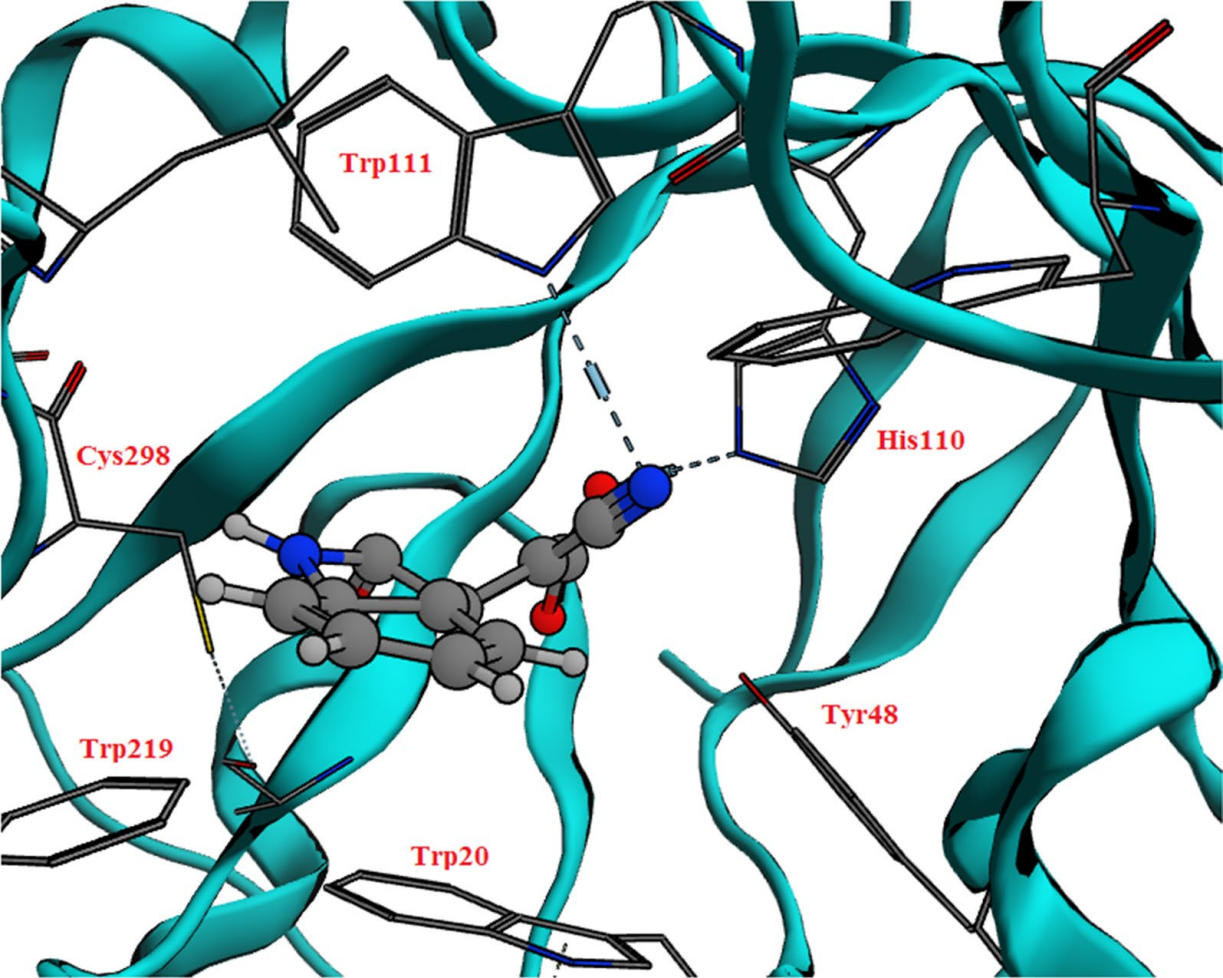
The predicted orientation mode of imidazolidine-2,5-dione ring of the spirohydantoin

A pre-molecular docking study was performed in order to compare the free energy of binding ΔG and the orientation of both spirohydantoin (scaffold B) and the (scaffold A). The results showed that the later showed higher ΔG (− 20.37 kcal/mol) while, spirohydantoin showed ΔG (− 17.54 kcal/mol). The imidazolidine-2,5-dione ring of the spirohydantoin showed only two hydrogen bonds when compared to the (scaffold A) (Fig. 6).

### Our proposed design

**Fig. 7:**
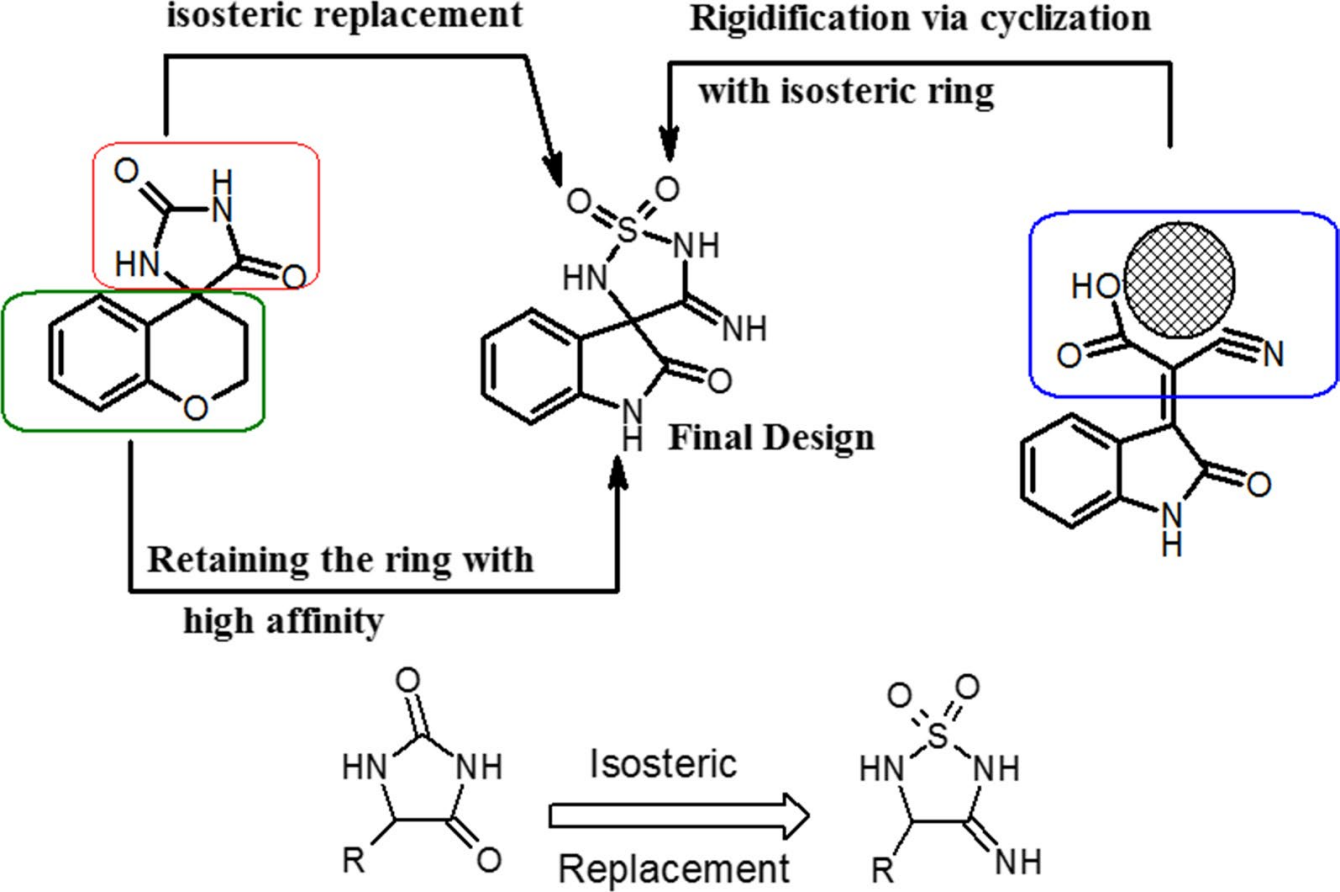
The design strategy of the proposed compounds

We tried to keep the 2-oxo-2,3-dihydroindol-3-yl ring with the high affinity and to apply an isosteric replacement of the imidazolidine-2,5-dione with imino-thiadiazolidine-1,1-dioxo scaffold to increase the pharmacodynamic interactions. The selection of this scaffold features more predicted interactions with the strong electron accepting sulfonyl group. In addition, the imino group that acts as an electron accepting that mimics the nitrile group in scaffold A. The imino-thiadiazolidine-1,1-dioxo ring is a rigid scaffold that will fit in a stable orientation because of its conformational restriction where it will have less number of proposed conformations when compared to the acyclic cyano(2-oxo-2,3-dihydroindol-3-yl) acetic acid moiety that is more flexible and has many conformations which can lead to less binding stability. This proposed design is an optimization of both the acetic acid derivatives and sorbinil scaffold aiming to get a highly potent novel agents (Fig. 7).

### Docking study of the proposed structures

**Fig. 8:**
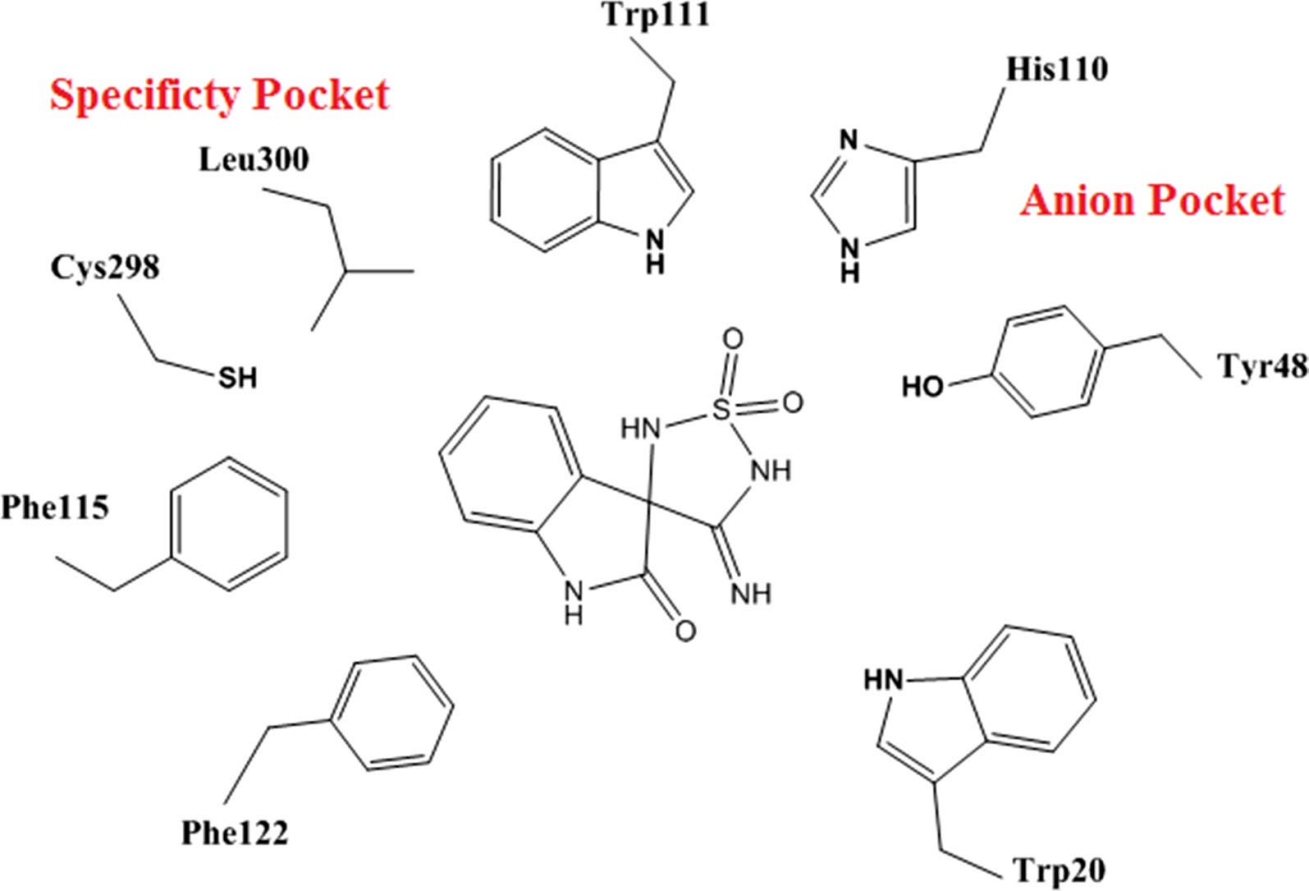
Possible fitting of the 4′-iminospiro[indoline-3,3′-[1,2,5] thiadiazolidinyl]-2-one 1′,1′-dioxide scaffold

**Fig. 9:**
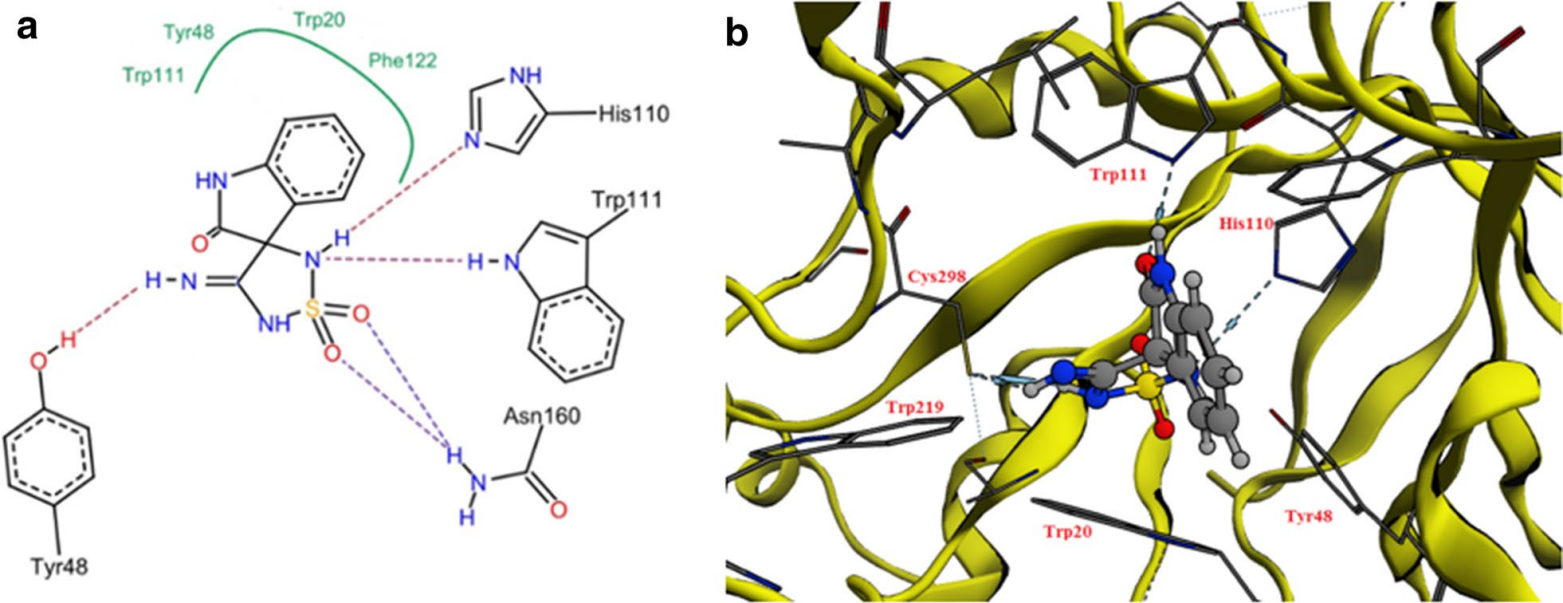
**a** 2D representation of the docking of proposed compound 1 showing possible interactions. **b** 3D orientation resulted from docking that confirmed the predicted binding

Nine compounds were designed that feature different substitutions at the indol ring to study the structure activity relationship of these substituents. Molecular docking study was conducted for 9 proposed compounds with indole—imino-thiadiazolidine-1,1-dioxo scaffold that share the proposed design in order to predict their mode of binding and compare it to the well-known AR inhibitors (Fig. 8). Two software; Leadit 2.1.2 and MOE 2016.08 were used to ensure the validity of the docking process. As per the docking results (Table 3) all compounds shared, the same pose in which the indole ring was positioned in a central position toward both the specificity and hydrophobic pockets. The sulfonyl group of the imino-thiadiazolidine-1,1-dioxo ring showed two hydrogen bonds with Asn160. The imino NH = formed a hydrogen bond (H–B) with the –OH of the Tyr48. His110 and Trp111 showed two hydrogen bonds with the –NH of the imino-thiadiazolidine-1,1-dioxo ring (Fig. 9).

**Table 3:** Molecular docking results by two programs; MOE and Leadit softwares

Compound	R =	Docking score (Leadit)	ΔG score MOE 2016.08	Lipophilic contribution score	Conformational entropy score	Predicted *p*IC_50_
1	H	− 26.32	− 23.40	− 6.70	1.40	6.838
2	5-Cl	− 26.62	− 23.37	− 7.80	1.40	6.967
3	5-Br	− 29.95	− 24.75	− 8.05	1.40	5.387
4	5-CH_3_	− 30.79	− 24.88	− 8.11	1.40	6.713
5	5-NO_2_	− 33.24	− 25.32	− 7.50	1.40	8.344
6	5-I	− 27.22	− 24.65	− 7.44	1.40	6.952
7	4,7-diCl	− 21.33	− 20.95	− 7.34	1.40	4.340
8	5,7-diCl	− 23.64	− 20.08	− 7.41	1.40	7.178
9	7-Cl	− 23.96	− 20.14	− 7.20	1.40	6.767

**Fig. 10:**
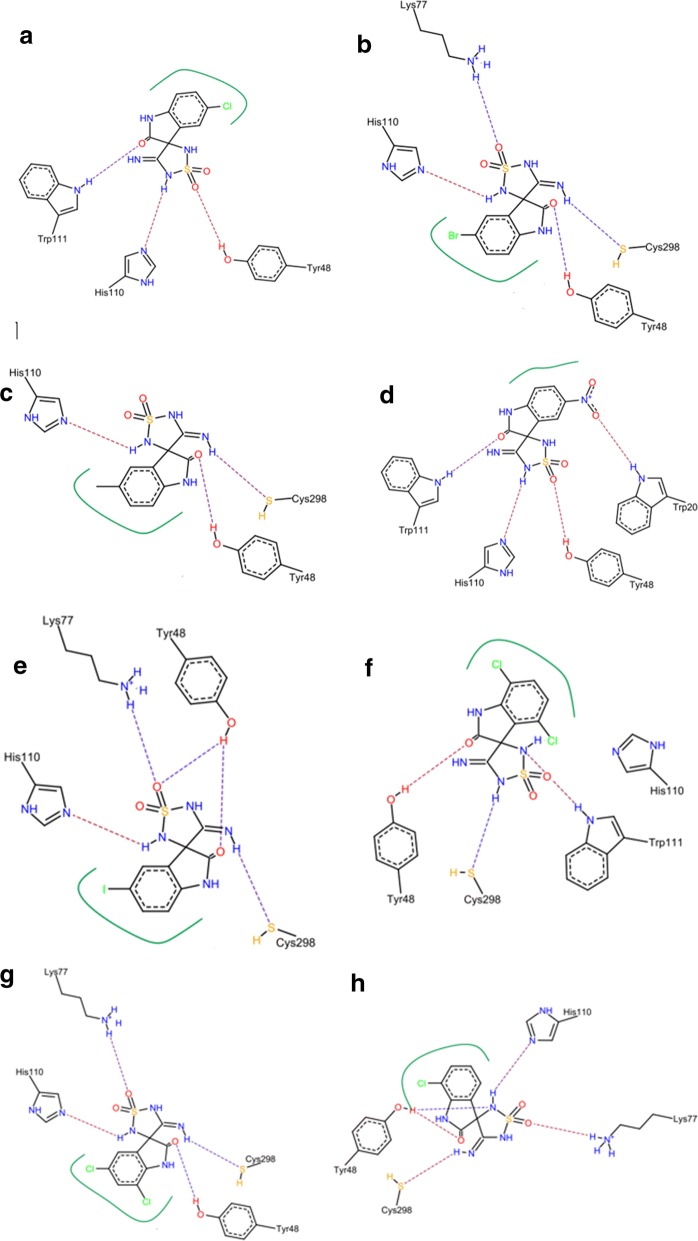
The predicted binding poses of compounds 2 (**a**), 3 (**b**), 4 (**c**), and 5 (**d**) showing the possible interactions. The predicted binding poses of compounds 5 (**e**), 7 (**f**), 8 (**g**), and 9 (**h**) showing the possible interactions

The docking results of compound 2 showed a H–B formed between the C=O of indole with Trp111 (NH). The sulfonyl group formed a strong H–B with the hydroxyl group of Tyr48. The imino-thiadiazolidine-1,1-dioxo scaffold contributed by one of its –NH to form a H–B with His110 (Fig. 10a).

Compound 3 contributed by three H–Bs, one formed between the sulfonyl group and NH of Lys77. The second was between the imine=NH and Cys298. The last one formed between the C=O and hydroxyl group of the Tyr48 (Fig. 10b). Compound 3 showed almost the same interactions of compound 2 with the same residues (Fig. 10c). On the other hand, compound 5 with 5-nitro substitution illustrated a strong H–B with –NH of Trp20. The sulfonyl group of compound 5 also contributed by another interaction with Tyr48. This compound was able to interact with four important residues such as Trp111, Trp20, His110 and Tyr48 at the same pose (Fig. 10d).

Compound 5 showed the top free energy of binding value (− 33.24 kcal/mol) among the other docked compounds when was docked with Leadit and it retained the same top rank with the second docking with MOE 2016.08 (− 25.32 kcal/mol) (Table 3). Compounds 6, 7, 8, and 9 showed the same interactions profile with Lys77, Tyr48, Cys298 and His110 (Fig. 10e–h).

### Molecular dynamic simulations

**Fig. 11:**
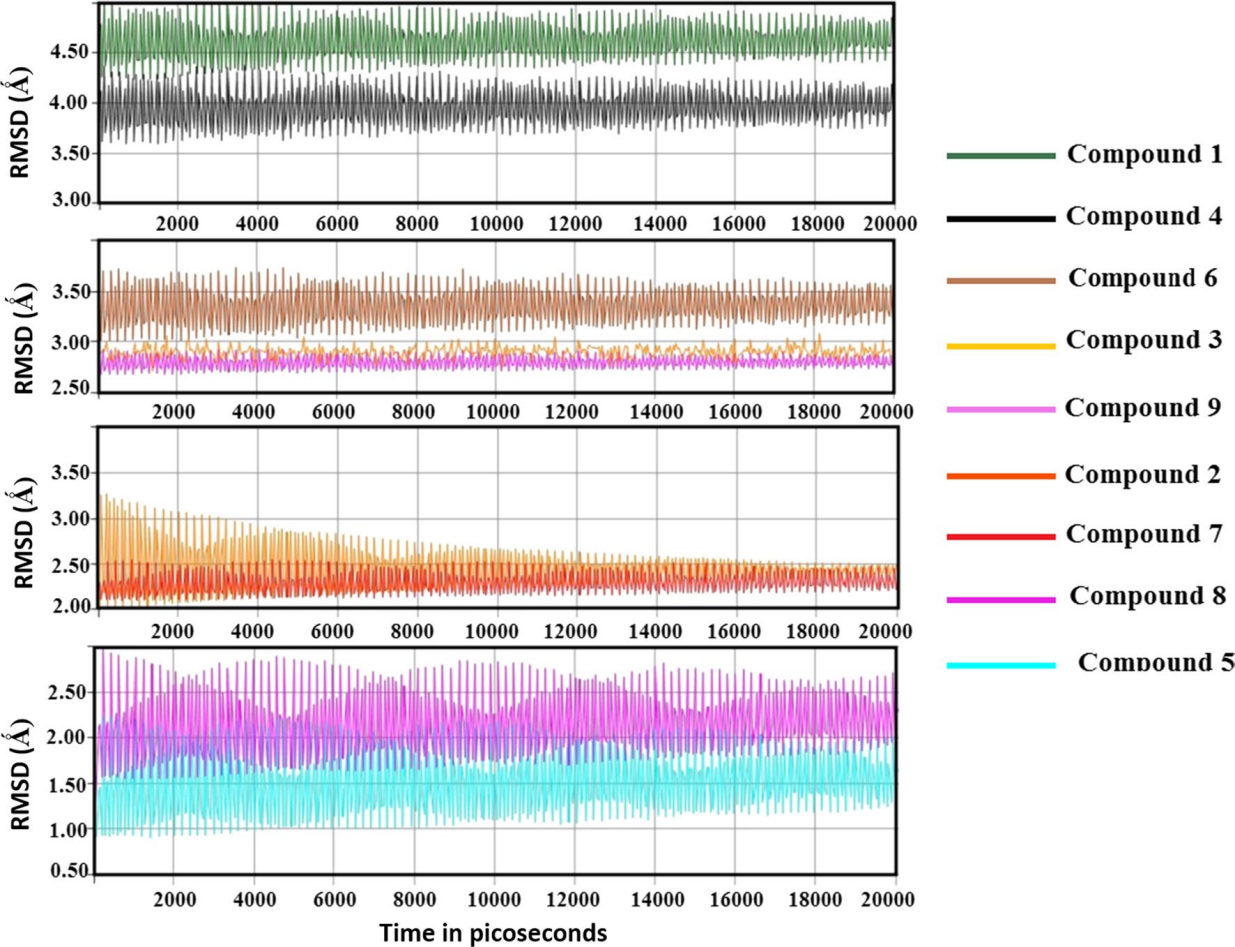
Molecular dynamic simulations showing the computed RMSD against time within 20,000 picoseconds

The main aim of molecular dynamic simulations was to evaluate the stability of binding of the proposed docked compounds inside the active side of AR during simulation time. The time period was 20,000 picoseconds (20 ns). The trajectory was analyzed each 1000 picoseconds. The root mean square deviation (RMSD) for each compound-protein complex was computed. According to the results, compound 5 with 5-nitro substitution achieved the lowest RMSD ranged from 1.00 to 1.50 which confirmed its strength of binding. The nitro group of this compound may contribute to the π–π hydrophobic interactions between the electron deficient environment of the phenyl moiety of indole ring that resulted from the nitro group substitution and the hydrophobic residues in the specificity pocket like; Leu300, Phe115 and Trp111. The second two compounds were compound 8 and 7 with the 5,7-dichloro and 4,7-dichloro substitution that showed RMSD values of 2.00 and 2.50 respectively. It seems that the electron deficiency environment that was created either by the electron withdrawing groups or halo atoms can support the stability of binding. The 5-chloro derivative showed RMSD of 3.00 that started to decrease gradually by time till reach a steady state at 2.00. The 7-chloro derivative (compound 9) and 5-bromo (compound 3) shared a common oscillation that appeared at RMSD of 2.70. Then the 5-iodo derivative appeared to have RMSD at 3.50. The absence of any substitution as in compound 1 was very clear to affect its RMSD that was 4.50. Also, the electron donating methyl group in compound 4 was high at RMSD of 4.00. Generally, the binding at both the specificity pocket and the hydrophobicity pocket may require a kind of π–π hydrophobic interactions that may be supported by either electron withdrawing groups or halo substitution (Fig. 11).

### Chemical synthesis of the proposed structures

**Scheme 1:**
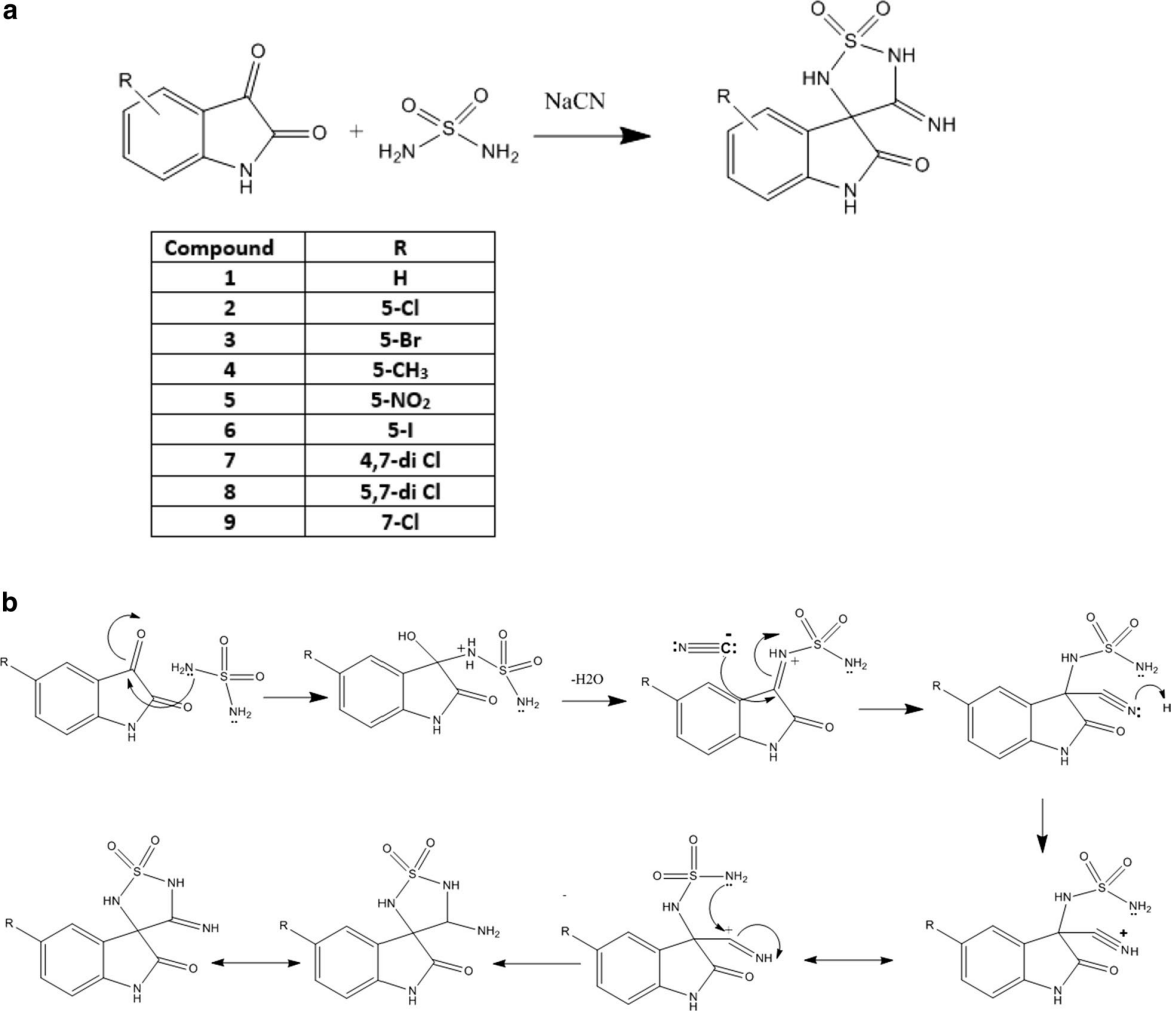
**a** The chemical synthesis pathway of the target compounds. **b** Proposed mechanism for the synthesis of the target compounds

The chemical synthesis of the new compounds was achieved according to the reported method [26]. The ketone functional group (C=O) in the isatin ring was highly active to react with sulfamide in presence of sodium cyanide in 70% ethanol to yield imino-4-substituted 1,2,5-thiadiazolidines (Scheme 1). The reaction is a simple one step reaction in which cyclization of the 5-membered 3-imino 1,2,5-thiazolidine 1,1-dioxide ring to give the final product. The most characteristic spectroscopic properties of the compounds are the IR absorption bands (1153) cm^−1^ and (1395) cm^−1^ for the sulfonyl group. The imino stretching band was found at 1650 cm^−1^.

The mechanism by which the reaction is performed is a modified Strecker synthesis, which was used for hydantoin synthesis (Scheme 1a). The reaction involves a Schiff’s base reaction between sulfamide and one of the C=O group of isatin. Then the cyanide anion will be added to the partially negative carbon of C=N, after this addition the newly formed nitrile derivative will be rearranged by resonance forming –C+=N– bond which will allow another addition to its positive carbon by the lone pair of electrons on the second free amino group in sulfamide. This will close the 3-imino-2,5-thiadiazoline-1,1-dione based scaffold (Scheme 1b).

### In vitro cytotoxic testing

**Fig. 12:**
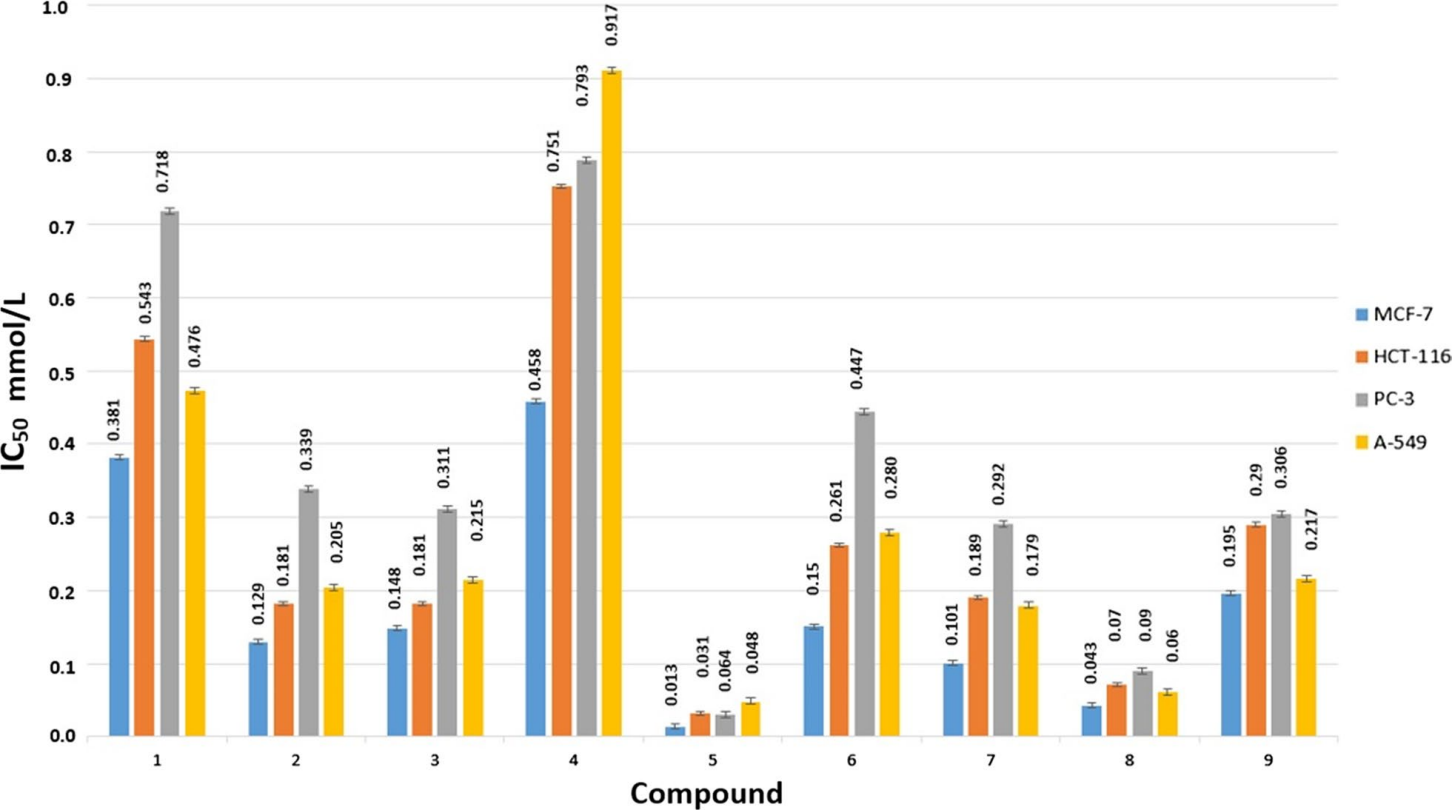
The in vitro anticancer activity (mmol/L) of the tested compounds against MCF-7, HCT-116, PC-3, and A-549 cell lines

**Fig. 13:**
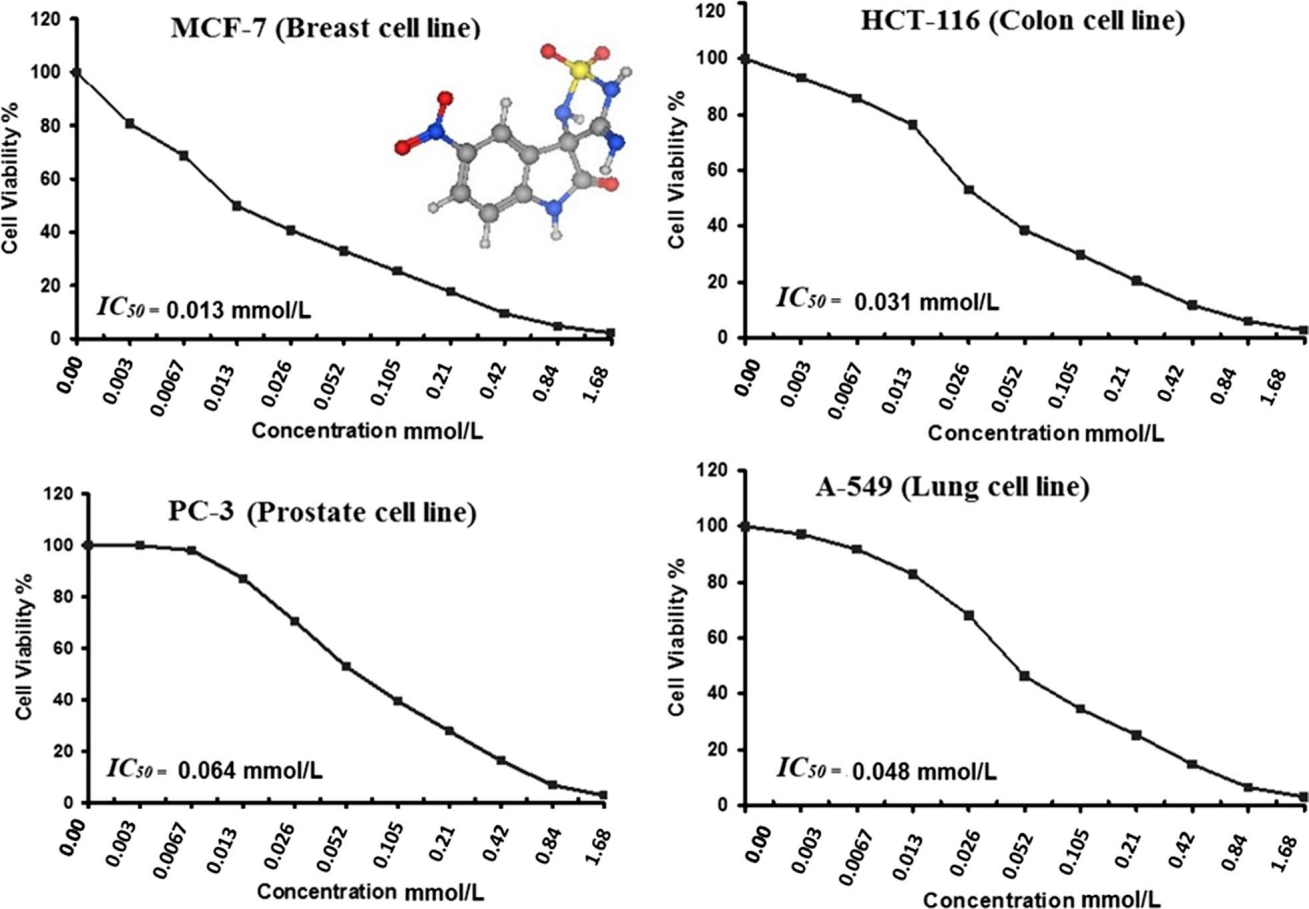
The IC_50_ values (mmol/L) of the most active compound 5 against MCF-7, HCT-116, PC-3, and A-549 cell lines

The anticancer activity of the newly designed compounds was evaluated against four cell lines; MCF-7, HCT-116, PC-3, and A-549. All compounds showed promising results especially compound 5 with the 5-nitro substitution that achieved the highest inhibition for all cell lines (Fig. 12). Compound 5 showed IC_50_ of 0.013, 0.031, 0.064, and 0.048 mmol/L against MCF-7, HCT-116, PC-3, and A-549 respectively (Fig. 13). The predicted ranking for the biological activity was almost ranked with most of the compounds. Compound 5 was the top ranked then both compound 8 and 7. Compound 1 that lacks any substitution and compound 4 with the electron donating methyl group showed the least cytotoxic effect.

## Conclusion

Aldose reductase enzyme was reported to have a relationship with cancer growth. The inhibition or deletion of aldose reductase contributed to the inhibition of the growth of cancer cells. From this concept, conformational restriction approach together with isosteric replacement approach were used to design novel nine substituted-4′-iminospiro[indoline-3,3′-[1,2,5] thiadiazolidinyl]-2-one 1′,1′-dioxide derivatives that were designed to target aldose reductase inhibition. In silico studies including; docking, dynamics, and QSAR-predictions confirmed the high free energy of binding, and binding stability of the tested compounds when compared to well-known aldose reductase inhibitors. In addition, compound 5 that achieved the highest inhibitory activities with IC_50_; 0.013, 0.031, 0.064, and 0.048 mmol/L against breast, colon, prostate, and lung cell lines respectively. Compound 5 is considered as a lead compound that may need more attention in future studies.

## Materials and methods

### In silico QSAR study

In the training set for each compound, a total of 236 descriptors (151 for 2D descriptors and 85 for 3D descriptors) which are already available in MOE 2016.08 package in the QSAR study were computed. The validation of the generated models was done by using the training set. Two types of descriptors were tested; 2D descriptors that consist of physical properties, atom and bond counts, subdivided surface area, pharmacophore feature descriptors and partial charges, Kier and Hall connectivity and Kapper shape indices. The second type includes 3D descriptors as; potential energy descriptors, surface area, volume, MOPAC, shape descriptors and conformation dependent charge descriptors. The significance of the models was obtained statistically by partial least square analysis (PLS). *pKi* values were calculated by applying the following equation

$$ pKi = \, - { \log }\left( {\text{Ki}} \right) $$

To validate the model power, the correlation coefficient (R^2^) and root mean square error (RMSE) values were calculated for all descriptors. MSE was computed by applying the following equation:

$$ MSE(\alpha_{0} ,\alpha ) = \frac{1}{W}\sum\limits_{i = 1}^{m} {w_{i} } \left[ {y_{i} - \left( {\alpha_{0} + \alpha^{T} x_{i} } \right)} \right]^{2} . $$

### Molecular docking

The crystal structure of human aldose reductase in complex with Fidarestat was downloaded from protein data bank (pdb code = 1ef3). X-ray crystallography method was used to obtain this protein with resolution of 2.8 Å and R-value free of 0.289. All coordinates were derived from protein data bank and all interactions were visualized between the conserved residues; Trp111, His110, Tyr48, Trp29, Trp219, Phe122, Phe115, Cys298, Leu300 and the complexed ligand. The MOE 2016.08 docking protocol was applied, in which the triangle method was used as a placement method with timeout of 300 s, and number of return poses as 1000. London dG was used as a rescoring method. Force field was selected as a refinement method by applying MMFF94x. While in case of leadit, the docking protocol involved loading the protein into Leadit 2.1.2 and the receptor components were chosen by selection of chain A as a main chain. Fidarestat was chosen as a reference ligand to which all coordinates were computed and this enabled the definition of the binding site. Amino acids within radius 6.5 Å were selected in the binding site. All chemical ambiguities of residues left as default. Ligand binding was computed by the classic Triangle matching method (enthalpy). The scoring default settings were kept. Intra-ligand clashes were computed by using clash factor = 0.6. Maximum number of solutions per iteration = 200. Maximum of solution per fragmentation = 200. The docking strategy was applied by using the base placement method.

### Molecular dynamics

MOE 2016.08 was used for computing the molecular dynamic simulations. The best pose from each docking process for each compound was kept inside the active site in order to be used for MD. The quality of the temperature-related factors, electron density, and protein geometries was tested. Energy minimization was computed after adding all the hydrogens. All solvent molecules within the system were deleted before solvation; salt atoms (NaCl) were added in 0.1 mol/L concentration to get a neutral biomolecular system. The solvent atoms (water) were added to surround the biomolecular system (protein–ligand complex) in a cube shape with cell dimensions; 81.9 × 81.9 × 81.9. The total number of molecules within the system was 16,475, 1.01 g/cm^3^. Amber 10:EHT was selected as a force field in the potential setup step. All Van der Waals forces, electrostatics, and restraints were enabled. The temperature of the system was increased from 0 to 300 K by adjusting the heat followed by equilibration and production for 300 ps; cooling was then initiated until 0 K was reached. The simulation was conducted over 20 ns period of time (20,000 ps).

### Chemical synthesis

#### General method for preparation of the target compounds

To a 70% aqueous ethanolic solution (20 mL) contains an appropriate isatin derivatives (10 mmol), we added sulfamide (20 mmol) and sodium cyanide (11 mmol). The resulted mixture was refluxed for 6 h and then evaporated to dryness using rotavapor. An aqueous solution of 1 N sodium hydroxide (10 mL) was added to the residue. The solution was washed with ethyl acetate (2 × 10 mL) and diethyl ether (10 mL). The aqueous layer was then acidified with 1 N hydrochloric acid solution, leading to the precipitation of a solid that was filtered, dried and recrystallized from ethanol.

#### 4′-iminospiro[indoline-3,3′-[1,2,5]thiadiazolidin]-2-one 1′,1′-dioxide (1)

Yellowish brown solid, Yield (68%). mp; 263 °C. IR γ_max_ cm^−1^ (KBr): 1115, 1335, 1630, 1700, 1550, 1600. ^1^H NMR (400 MHz, DMSO-*d*6) δ: 6.45 (s, 1H), 6.60 (s, 1H), 6.92 (d, *J* = 7.78 Hz, 1H), 7.25 (s, 1H), 7.35 (t, *J *= 8.02 Hz, *1H*), 7.40 (t, *J *= 8.02 Hz, *1H*), 8.10 (d, *J *= 7.68 Hz, 1H), 11.01 (s, 1H).

#### 5-Chloro-4′-iminospiro[indoline-3,3′-[1,2,5]thiadiazolidin]-2-one 1′,1′-dioxide (2)

Dark red solid, Yield (62%). mp; 271 °C. IR γ_max_ cm^−1^ (KBr): 1120, 1350, 1638, 1700, 1550, 1600. ^1^H NMR (400 MHz, DMSO-*d*6) δ: 6.48 (s, 1H), 6.60 (s, 1H), 7.26 (s, 1H), 7.46 (dd, *J* = 1.9 Hz, *J* = 8.5 Hz, 1H), 7.58 (d, *J* = 8.5 Hz, 1H), 8.07 (d, *J* = 1.9 Hz, 1H), 10.97 (s, 1H).

#### 5-Bromo-4′-iminospiro[indoline-3,3′-[1,2,5]thiadiazolidin]-2-one 1′,1′-dioxide (3)

Brown solid, Yield (55%). mp; 268 °C. IR γ_max_ cm^−1^ (KBr): 1122, 1348, 1650, 1710, 1550, 1650. ^1^H NMR (400 MHz, DMSO-*d*6) δ: 6.50 (s, 1H), 6.68 (s, 1H), 6.82 (d, *J* = 8.24 Hz, 1H), 7.30 (s, 1H), 7.54 (dd, *J* = 1.92 Hz, *J* = 8.24 Hz, 1H), 8.25 (d, *J* = 1.92 Hz, 1H), 11.00 (s, 1H).

#### 5-Methyl-4′-iminospiro[indoline-3,3′-[1,2,5]thiadiazolidin]-2-one 1′,1′-dioxide (4)

Red solid, Yield (70%). mp; 280 °C. IR γ_max_ cm^−1^ (KBr): 1117, 1336, 1640, 1705, 1550, 1600. ^1^H NMR (400 MHz, DMSO-*d*6) δ: 2.30 (s, 3H), 6.50 (s, 1H), 6.54 (s, 1H), 6.87 (d, *J* = 8.00 Hz, 1H), 7.23 (s, 1H), 7.40 (d, *J* = 7.84 Hz, 1H), 7.66 (s, 1H), 11.10 (s, 1H).

#### 5-Nitro-4′-iminospiro[indoline-3,3′-[1,2,5]thiadiazolidin]-2-one 1′,1′-dioxide (5)

Reddish brown solid, Yield (73%). mp; 278 °C. IR γ_max_ cm^−1^ (KBr): 1125, 1355, 1642, 1700, 1565, 1336. ^1^H NMR (400 MHz, DMSO-*d*6) δ: 6.62 (s, 1H), 6.70 (s, 1H), 7.05 (d, *J* = 8.73 Hz, 1H), 7.35 (s, 1H), 8.22 (d, *J* = 8.73 Hz, 1H), 8.75 (s, 1H), 11.53 (s, 1H).

#### 5-Iodo-4′-iminospiro[indoline-3,3′-[1,2,5]thiadiazolidin]-2-one 1′,1′-dioxide (6)

Yellowish white solid, Yield (69%). mp; 282 °C. IR γ_max_ cm^−1^ (KBr): 1123, 1345, 1635, 1700, 1550, 1600. ^1^H NMR (400 MHz, DMSO-*d*6) δ: 6.60 (d, *J *= 8.1 Hz, 1H), 6.65 (s, 1H), 6.72 (s, 1H), 7.55 (d, *J* = 8.1 Hz, 1H), 7.40 (s, 1H), 7.71 (s, 1H), 10.53 (s, 1H).

#### 4,7-Dichloro-4′-iminospiro[indoline-3,3′-[1,2,5]thiadiazolidin]-2-one 1′,1′-dioxide (7)

Orange solid, Yield (58%). mp; 265 °C. IR γ_max_ cm^−1^ (KBr): 1115, 1335, 1632, 1702, 1550, 1600. ^1^H NMR (400 MHz, DMSO-*d*6) δ: 6.60 (s, 1H), 6.68 (s, 1H), 6.99 (d, *J* = 8.4 Hz, 1H), 7.25 (d, *J* = 8.4 Hz, 1H), 7.33 (s, 1H), 11.97 (s, 1H).

#### 5,7-Dichloro-4′-iminospiro[indoline-3,3′-[1,2,5]thiadiazolidin]-2-one 1′,1′-dioxide (8)

Dark orange solid, Yield (64%). mp; 272 °C. IR γ_max_ cm^−1^ (KBr): 1118, 1342, 1638, 1710, 1550, 1600. ^1^H NMR (400 MHz, DMSO-*d*6) δ: 6.60 (s, 1H), 6.62 (s, 1H), 7.23 (s, 1H), 7.47 (d, *J* = 2.01 Hz, 1H), 7.91 (d, *J* = 2.01 Hz, 1H), 11.37 (s, 1H).

#### 7-Chloro-4′-iminospiro[indoline-3,3′-[1,2,5]thiadiazolidin]-2-one 1′,1′-dioxide (9)

Dark yellow solid, Yield (70%). mp; 257 °C. IR γ_max_ cm^−1^ (KBr): 1120, 1350, 1638, 1700, 1550, 1600. ^1^H NMR (400 MHz, DMSO-*d*6) δ: 6.45 (s, 1H), 6.58 (s, 1H), 6.90 (d, *J *= 8.0 Hz, 1H), 7.20 (s, 1H), 7.24 (d, *J* = 8.0 Hz, 1H), 7.34 (d, *J* = 8.0 Hz, 1H), 10.99 (s, 1H).

### In vitro anticancer evaluation

The cytotoxicity assay was performed against breast cancer MCF-cell line and Lung cancer A-549 cell lines. This was done by seeding the cells in 96-well plate at a cell concentration of 1 × 10^4^ cells per well in 100 µL of growth medium. The next step involved the addition of different concentrations of the tested sample. A multichannel pipette was used to dispense two-fold dilutions of the tested synthesized compound to a confluent cell monolayers distributed into 96-well (Falcon, NJ, USA). Then incubated at 37 °C in a humidified incubator with 5% CO_2_ for 48 h. Three wells were used for each concentration of the tested sample. Control cells were performed without test sample and with or without DMSO (0.1%). After incubation. Various concentrations of sample were added, and incubated for 24 h. A colorimetric method was used to determine the viable cells yield. The media were aspirated and the crystal violet solution (1%) was added. Tap water was used to rinse the plates after removing the stain. Glacial acetic acid (30%) was then added to all wells and mixed. The plates were gently shaken on Microplate reader (TECAN, Inc.), and then the absorbance of the plates were measured at a wavelength of 490 nm. All results were corrected for background absorbance detected in wells without added stain. Treated samples were compared with the cell control in the absence of the tested compounds. All experiments were carried out in triplicate. The cell cytotoxic effect of each tested concentration was calculated. A microplate reader (SunRise, TECAN, Inc, USA) measured the optical density to determine the number of viable cells. The percentage of viability was calculated as [1 − (ODt/ODc)] × 100% where ODt is the mean optical density of wells treated with the tested sample and ODc is the mean optical density of untreated cells. The survival curve of each tumor cell line after treatment was obtained by plotting the relation between surviving cells and drug concentration. The 50% inhibitory concentration (IC_50_), the concentration required to cause toxic effects in 50% of intact cells, was estimated from graphic plots of the dose response curve for each conc. using Graphpad Prism software (San Diego, CA. USA). The biological evaluation was done at the Regional Center for Mycology & Biotechnology, Al-Azhar University.

## Abbreviations

AR: aldose reductase
QSAR: quantitative structure activity relationship
MOPAC: molecular orbital package
RMSE: root of mean square error
RMSD: root of mean square deviation
PDB: protein data bank
Trp: tryptophan
His: histidine
Tyr: tyrosine
Phe: phenyl alanine
Cys: cysteine
Leu: leucine
MOE: molecular operating environment
Asn: asparagine
MCF-7: human breast adenocarcinoma cell line
HCT: colon cancer cell line
PC-3: prostate cancer cell line
A-549: lung cancer cell line
PLS: partial least square analysis
2D: 2 dimensional
3D: 3 dimensional
Ps: picoseconds
DMSO: dimethyl sulfoxide
